# Chasing intermediate hosts of *Echinococcus multilocularis* at the southern edge of its European distribution using red fox stomach content analysis

**DOI:** 10.1016/j.ijppaw.2025.101095

**Published:** 2025-06-02

**Authors:** Cafiero Salvatore Andrea, Cenni Lucia, Rossi Chiara, Pascoe Emily Louise, Obber Federica, Da Rold Graziana, Citterio Carlo Vittorio, Casulli Adriano, Hauffe Heidi Christine, Massolo Alessandro

**Affiliations:** a Ethology Unit, Department of Biology, University of Pisa, Pisa, Italy; b Conservation Genomics Research Unit, Centre for Research and Innovation, Fondazione Edmund Mach, San Michele All’Adige, Italy; c Istituto Zooprofilattico Sperimentale delle Venezie – Legnaro (PD), Italy; d European Union Reference Laboratory for Parasites (EURLP), Department of Infectious Diseases, Istituto Superiore di Sanità, Rome, Italy; e WHO Collaborating Centre for the Epidemiology, Detection and Control of Cystic and Alveolar Echinococcosis, Department of Infectious Diseases, Istituto Superiore di Sanità, Rome, Italy; f National Biodiversity Future Centre (NBFC), Palermo, Italy; g Faculty of Veterinary Medicine, University of Calgary, Calgary, AB, Canada; h Chrono-environnement (UMR 6249), CNRS, Université Marie et Louis Pasteur, F-25000, Besançon, France

**Keywords:** Italy, *Vulpes vulpes*, *Microtus arvalis*, Rodent, Diet, Molecular screening

## Abstract

*Echinococcus multilocularis* (*Em*) is a Taeniidae cestode circulating among canids (definitive hosts) and among voles (intermediate hosts). Humans can develop alveolar echinococcosis (AE) following egg ingestion. In Italy, *Em* is present in red foxes (*Vulpes vulpes*) from the Trentino-Alto Adige/Südtirol Region, with reports in canids from Liguria (wolf, *Canis lupus*, and domestic dog, *Canis lupus familiaris*) and Tuscany (wolf and red fox) Regions. The first autochthonous case of human AE was recently confirmed in the Province of Bolzano. Despite its relevance, the intermediate hosts maintaining the *Em* life cycle in this area have never been identified.

This study aimed to fill this knowledge gap by collecting ingested rodents from the stomachs of 148 legally culled or found dead foxes across the Province of Bolzano. For 142 prey items, species was ascertained from tissue DNA using a PCR of a 350 bp fragment of *cytb* mitochondrial gene. Positivity to *Em* was investigated by RT-PCR and conventional PCR on organ tissues from 97 rodents targeting a 69 bp fragment of *cob* and a 126 bp fragment of *nad2* mitochondrial genes, respectively.

*Microtus arvalis* was the most common prey rodent in terms of both frequency in foxes (20.8 %; 30/144) and total rodent prey items (81.7 %; 116/142). Other prey species included nine *Arvicola amphibius*, five *Microtus lavernedii*, two *M*. *subterraneus*, one *M*. *liechtensteini*, six *Clethrionomys glareolus*, one *Apodemus flavicollis* and two *Ap*. *sylvaticus*. Only 3/97 rodents were *Em*-positive (*M. arvalis*; 3/85) in two *Em*-positive red foxes. Prevalence in *M. arvalis* was estimated at 0.035 (95 % CI: 0.008–0.103). Further research is required to explain why *Em* distribution is limited to this area in the eastern Italian Alps despite the spatial contiguity to hyperendemic foci.

## Highlights

• Most red fox rodent prey are susceptible intermediate hosts of *Echinococcus multilocularis* in this marginal area.
• *Microtus arvalis* was the most frequent fox rodent prey and the only rodent species positive for *Echinococcus multilocularis*.
• Analysis of stomachs of definitive hosts is a valid approach for screening *Echinococcus multilocularis* in canid rodent prey.
• Molecular analyses are useful to detect parasite DNA in organs without macroscopic lesions.

## Graphical abstract

**Figure:**
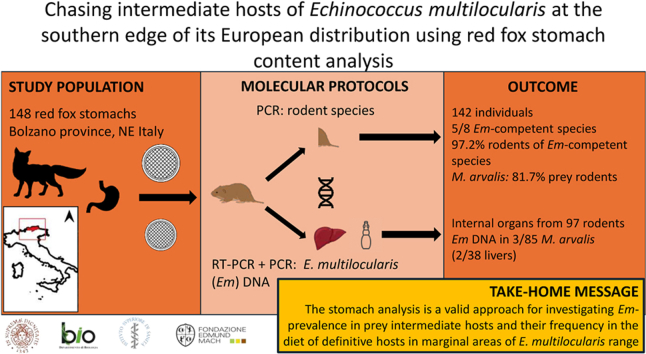

## 1 Introduction

Alveolar echinococcosis (AE) is a neglected zoonosis caused by the larval stage of the cestode *Echinococcus multilocularis* (*Em*) across the northern hemisphere (Deplazes et al., 2017). Its predator-prey life cycle is mainly sylvatic, and involves small rodents, especially voles, as intermediate hosts (IHs) and wild/domestic canids, especially red foxes (*Vulpes vulpes*), as definitive hosts (DHs) (Romig et al., 2017). Humans acquire infection after the ingestion of eggs excreted in the faeces of DHs, acting as accidental dead-end hosts (Conraths and Deplazes, 2015). If untreated, AE can lead to mortality in more than 90 % of cases within 10–15 years (Torgerson et al., 2010). Egg survival in the environment and the competent host habitat are strongly affected by winter temperature and humidity, annual rainfall and availability of high elevation pastures/grasslands (Veit et al., 1995; Giraudoux et al., 2003; Piarroux et al., 2015)*.* On a global scale, climate change, urbanisation and globalisation are the main parameters influencing host distribution and *Em* prevalence (Davidson et al., 2012; Atkinson et al., 2013; Liccioli et al., 2015), whereas at a local level the transmission pattern seems to be affected by spatial-temporal overlap between predator and prey, as well as the proportion of suitable IHs in the diet of DH predators (Liccioli et al., 2014; Raoul et al., 2015).

In the early 2000s an autochthonous focus of *Em* was discovered in red foxes from the Trentino-Alto Adige/Südtirol Region in northeastern Italy (Manfredi et al., 2002; Casulli et al., 2009), where prevalence has reached 2.9 % and 12.6–14.3 % in the Provinces of Trento (Casulli et al., 2005) and Bolzano (Citterio et al., 2021; Obber et al., 2022), respectively. In the latter, *Em* was opportunistically detected in four rodent species, namely *Arvicola amphibius*, *Apodemus sylvaticus*, *Eliomys quercinus* and *Rattus rattus* (Sgubin et al., 2022). However, their relevance in the red fox diet is still unknown. Recently, *Em* eggs were detected in faeces of canids from the Maritime Alps (grey wolves and shepherd dogs) in the Liguria Region, bordering France (Massolo et al., 2018), and from the Apuan Alps (grey wolves and red foxes) in the Tuscany Region, central Italy (Cafiero et al., 2025). Moreover, the westward expansion of a new competent DH (golden jackal, *Canis aureus*) could alter local trophic network and, consequently, parasite transmission (Tsunoda and Saito, 2020; Lange et al., 2021; Torretta et al., 2021).

The reason why *Em* has not spread southward from the Italian Alps, and the role of small rodent assemblages in these and other marginal zones, have yet to be understood (Oksanen et al., 2016; Romig et al., 2017). Nonetheless, some authors proposed that the presence or absence of specific rodent species, such as common voles (*Microtus arvalis*), might represent the main limiting factor to the parasite spread (Guerra et al., 2014). However, other arvicolines, such as field voles (*M*. *agrestis*) and East European grey voles (*M*. *levis*) can also play a key role as IHs in marginal areas of the *Em* European range (Stien et al., 2010; Miller et al., 2016). In addition, *Em* spillover from known to new host species has been documented (Liccioli et al., 2013; Chaignat et al., 2015).

*Echinococcus multilocularis* prevalence in DHs was proven to be modulated by spatial and temporal overlap with *Em*-infected prey (Robardet et al., 2011; Liccioli et al., 2014). However, detecting *Em* in wild rodents by field trapping is very challenging and time consuming, leading to very low estimated prevalences (Miller et al., 2016; Gürler et al., 2023), even in hyperendemic regions (Oksanen et al., 2016). Cyclic fluctuations and outbreaks of vole populations (Tkadlec and Zejda, 1998; Jacob et al., 2020), trapping technique efficiency and behavioural or environmental differences could lead to an underestimation of infection status, although some authors suggested a role of *Em* in facilitating vole predation by DHs (Vervaeke et al., 2006), balancing the above-mentioned issues. Despite a number of studies on parasitic manipulation of host behaviour (Lefèvre et al., 2009; Thomas et al., 2010; Lafferty and Shaw, 2013), this hypothesis could not be tested for *Em* until recently, when a pilot study revealed a significant increase of risk-taking behaviour in infected common voles, like feeding frequency and above-bedding activity (Martini et al., 2024). These effects on infected prey behaviour support the manipulation hypothesis which implies a higher likelihood of predation of infected prey and likely affects *Em* transmission dynamics.

The proportion of competent hosts within the prey community was also reported as a relevant biotic factor affecting the *Em* distribution range (Botero-Cañola et al., 2019) and prevalence in DHs (Mori et al., 2023), focusing on the overall prey range rather than a single prey species. This ratio could differ in DHs diet due to complex ecological interactions (Raoul et al., 2010; Baudrot et al., 2016) and potential dilution effect depending on available prey biodiversity (Civitello et al., 2015) or alternative food items (Robardet et al., 2008). Moreover, the diet of wild canids can vary greatly across endemic regions (Kauhala and Kowalczyk, 2012; Soe et al., 2017; Lanszki et al., 2022) in accordance with locally available small mammal assemblages (Raoul et al., 2015; Mori et al., 2019). In addition, the current climate and landscape changes may alter the habitat of host communities, especially at the edges of their distribution (Warburton and Blanar, 2021), and subsequently their susceptibility to parasites (Atkinson et al., 2013).

In order to avoid the critical issues of detection of infections in IHs - usually carried out by rodent field-trapping (Sikó Barabasi et al., 2011; Miller et al., 2016; Gürler et al., 2023) - in areas at relatively low *Em* prevalence, sampling directly from the stomach content of DHs could be a more efficient and appropriate method for assessing the frequency of encounter between IH hosts and the parasite, as well as identifying which prey species might actually have a predominant role in transmission.

Through stomach content analysis one can simultaneously acquire both the proportion of competent IHs preyed upon by DHs, while increasing the likelihood of detecting parasitic infections within the prey assemblage, particularly in areas at relatively low *Em* prevalence in DHs, and even lower in IHs.

Therefore, this study thus aimed to enrich the body of data concerning the presence of *Em* in the small mammal assemblage in the Province of Bolzano, a southern marginal zone of the *Em* range in the Italian Alps, from which, unexpectedly, the parasite has not spread during the last two decades (Oksanen et al., 2016; Obber et al., 2022). Specifically, we aimed to 1) investigate the proportion of known competent IH rodent species in the red fox diet upon locally-present rodent assemblage, and 2) assess the prevalence of *Em*-infected rodents in a well-established sylvatic cycle in a marginal endemic area.

## 2 Materials and methods

### 2.1 Study area and sample collection

**Fig. 1:**
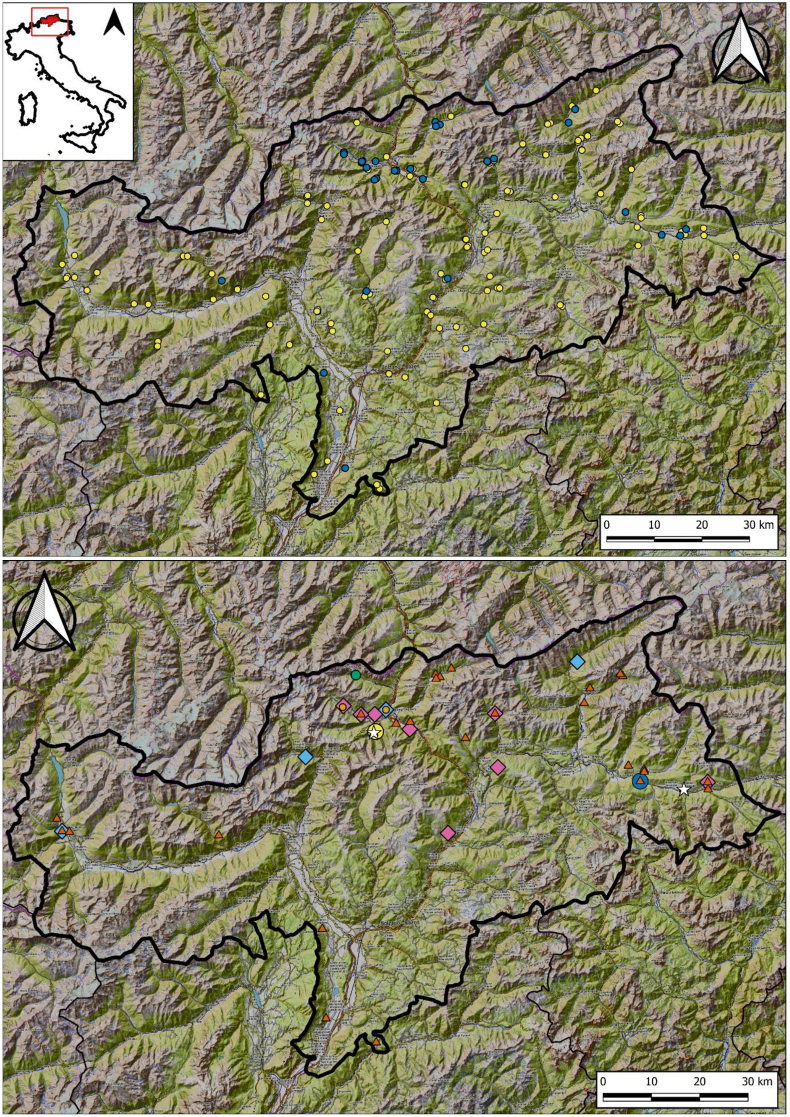
*Upper panel.* Distribution of red foxes culled across the Province of Bolzano, northeastern Italy, in 2019–2020 for which stomachs were available for examination of their gastric content to identify potential intermediate hosts (IH) for *Echinococcus multilocularis*. Regional boundaries are indicated in black, with the Province of Bolzano shown with a bolder boundary outline. Yellow and blue dots represent red foxes that resulted negative and positive to *E. multilocularis*, respectively. *Lower panel.* Distribution of prey rodent species in red fox diet. *Microtus arvalis* (brown triangles), *Arvicola amphibius* (pink diamonds), *Clethrionomys glareolus* (light blue diamonds), *M*. *subterraneus* (orange dots), *M*. *lavernedii* and *M*. *liechtensteini* (blue circle), *Apodemus sylvaticus* (light green dots) and *Ap*. *flavicollis* (yellow circle). The locations of stomachs containing *Em*-positive *M. arvalis* are represented by white stars.

We analysed the stomachs of red foxes legally culled by gamekeepers and authorized hunters, or found dead from passive surveillance, in hunting districts across the Province of Bolzano (Fig. 1) between July 2019 and January 2020. Whole carcasses were frozen at −80 °C for at least five days to deactivate *Em* eggs (following Veit et al., 1995), before being transferred to −20 °C for longer term storage. *Echinococcus multilocularis* infection in these foxes had been previously assessed (Obber et al., 2022). Necropsies for the sampling of different organs were performed at the Istituto Zooprofilattico Sperimentale delle Venezie (IZSVe). Each fox was georeferenced in the frame of the surveillance and research activities of IZSVe. Stomachs were then stored and delivered frozen to the laboratory of the Ethology Unit, Department of Biology, University of Pisa, where all the procedures described below were carried out.

### 2.2 Stomach content analysis

Each stomach was thawed overnight the day before analysis. The stomach contents were washed, filtered and macroscopically analysed with minor modifications of a well-established protocol (Kruuk and Parish, 1981; Balestrieri et al., 2011). Stomachs were longitudinally opened, emptied and washed with 1 L water into a graduated beaker, to estimate gastric content volume. The resulting volume was then filtered through three decreasing mesh size sieves (1,000, 225 and 75 μm). The residuals of the materials collected by the first two sieves were checked for dietary items.

We sorted items to the lowest taxon possible according to photographic galleries and dichotomous keys (Teerink, 1991; De Marinis and Agnelli, 1993; De Marinis and Asprea, 2006; Paolucci and Bon, 2022). Microscopic fragments were not included. Thus, the following categories were used for diet assessment: plant material, invertebrates, birds, small mammals (rodents, lagomorphs and insectivores), small carnivores (mustelids and felids), ungulates, undetermined mammals and anthropogenic material; all the remaining material was labelled as ‘undefined’. Frequency of occurrence (FO), i.e. the proportion of non-empty stomachs containing each item, mean volume in ml and estimated percentage volume (Loveridge and Macdonald, 2003) were calculated. Finally, the residuals from the last sieve were washed with demineralized water and pipetted into 1.5 ml eppendorfs. A small aliquot of the residual (10 ml) was investigated for the presence of *Em* protoscoleces under a light microscope, whereas all remaining residual material was left for DNA extraction and molecular detection of *Em*.

### 2.3 Rodents and lesions investigation

When present, we dissected both the abdomen and thorax of each rodent prey to collect organs for further examination of *Em* lesions using a stereomicroscope (Nikon, SMZ745T, 5x). We collected posterior legs and tails for molecular species identification from 143 rodents and we sampled internal organs and liquid from abdominal scraping for *Em* molecular screening, including 47 livers, from 97 individual rodents. Kidneys, lungs and hearts were also collected, since, although AE primarily affects the liver (Romig et al., 2017), *Echinococcus* spp. lesions have been occasionally reported in secondary locations (Okamoto et al., 1992; Geigy et al., 2013; Oikawa et al., 2013; Brunet et al., 2015). For every rodent with unrecognisable abdominal content, the abdominal walls were scraped with a scalpel and the resulting liquid was added to internal organs for *Em* molecular screening. Besides whole and digested carcasses, the minimum number of individual (‘MNI’) rodent prey for species identification was estimated based on the number of tails detected. Each sample was stored at −20 °C.

### 2.4 Molecular analyses

All the molecular protocols were implemented in the Animal, Environmental and Antique DNA Platform of the Conservation Genomics Research Unit, Centre for Research and Innovation, Fondazione Edmund Mach (FEM).

### 2.5 DNA extraction

DNA was extracted from 30 mg of tissue (pooled from different lobes in the case of livers, from several subsamples of other organs, and from the muscle of tails/legs) through an automated magnetic bead-based DNA extraction (Santa et al., 2019). The Mag-Bind Blood & Tissue DNA HDQ 96 kit (Omega Bio-Tek, Inc., Narcross Georgia 30071 USA) was used with a final elution of 150 μL according to the manufacturer's instructions. For each 96-well plate, 4–5 negative controls for DNA extraction were included to monitor any potential cross-contamination. Extracted DNA was stored at −20 °C.

### 2.6 Prey species classification

A conventional PCR (cPCR) was run to amplify a 350 bp fragment of cytochrome B (*cytb*) mitochondrial gene by forward cytb_H427 (5′-TCAGAATGATATTTGTCCTCA-3′) (Kocher et al., 1989) and reverse cytb_L79 (5′-AACATCTCAGCATGATGAA-3′) primers. After an initial denaturation at 94 °C for 2′, 40 PCR cycles consisted in 30’’ at 94 °C of denaturation, 30’’ at 52 °C of annealing and 1′ at 72 °C of extension, with a final extension at 72 °C for 5’. Reaction volume of 25 μl consisted in 13.25 μl of H_2_O, 5 μl of Flexi-Buffer, 2.5 μl of 25 mM MgCl_2_, 0.5 μl of each 10 pmol/μl primer, 0.5 μl of 10 mM dNTPs, 0.25 μl of 5 U/μL GoTaq polymerase and 2.5 μl of DNA template. Negative controls for both DNA extraction and PCRs were included to check for the presence of cross-contamination. Resulting PCR products were checked through the QIAxcel Advanced System (Qiagen, Germany) and then submitted for Sanger sequencing at the Sequencing and Genotyping Platform, FEM. Nucleotide sequences were analysed with the Basic Local Alignment Search Tool (BLAST; https://blast.ncbi.nlm.nih.gov/Blast.cgi) on NCBI (National Centre for Biotechnology Information) platform.

### 2.7 *Echinococcus multilocularis* in rodent prey

A Real-Time PCR kit (QuantiNova Pathogen + IC Kit, Qiagen, Germany) was used according to the manufacturer's instructions with minor modifications and 2 μl of DNA template. The RT-PCR was performed with ViiA7™ Real-Time PCR System, ThermoFisher Scientific, Waltham, MA. Thermal reactions consisted in RT-step at 50 °C for 10′, PCR activation at 95 °C for 2′ and 45 cycles of denaturation at 95 °C for 5’’ plus a combined annealing/extension at 58 °C for 30’’. A Cycles threshold (Ct) value of 40 was adopted as the cut off value. Both primers and probe targeting a 69 bp fragment of *cob* gene were used (Massolo et al., 2021). Each sample was analysed twice in the same 96-well plate. A synthetic DNA fragment with related primers and probe was part of the kit reagents and was added in each well (1 μl), besides the negative controls, for monitoring the goodness of the amplification. Extraction-internal controls and negative controls (demineralized water) were included to exclude cross-contamination during either DNA extraction or RT-PCR reaction, respectively.

A cPCR was also run targeting a 126 bp fragment of the mitochondrial *nad2* gene using *Em*-specific primers (Santa et al., 2019). The protocol consisted of an initial denaturation step at 95 °C for 2′, followed by 40 PCR cycles (15’’ at 95 °C denaturation, 25’’ at 58 °C annealing and 45’’ at 72 °C extension), with a final extension at 72 °C for 5’. A reaction volume of 45 μl included 26.75 μl of H_2_O, 10 μl of Flexi-Buffer, 5 μl of 25 mM MgCl_2_, 1 μl of each 10 pmol/μl primer, 1 μl of 10 mM dNTPs, 0.25 μl of 5 U/μL GoTaq polymerase and 5 μl of DNA template. Reaction products were screened using the QIAxcel Advanced System and amplicon products of approximately 126 bp were sent for Sanger sequencing.

### 2.8 Statistical analyses

Apparent prevalence (AP) and 95 % Confidence Intervals (CI) were estimated with the Jeffreys method (Brown et al., 2001) on EpiTools (Sergeant, ESG, 2018. Epitools Epidemiological Calculators. Ausvet. Available at: http://epitools.ausvet.com.au) and in R (version 4.4.1) by using package ‘binom’ and function ‘binom.confint’.

## 3 Results

Only four fox stomachs out of 148 analysed were completely empty. For the remaining 144 stomachs, the volume of gastric content ranged from less than 1–800 ml (mean ± SE = 95.7 ± 9.1 ml; median = 60 ml). For six stomachs, the contents had already been examined, and rodent remains had already been preliminarily isolated at the Istituto Zooprofilattico Sperimentale of Bolzano, Italy. Whole or parts of small mammals were present in 47 stomachs (FO 32.6 %). With regard to the other diet categories, the most common were vegetal and plant-related items (88.9 %), followed by invertebrates (27.8 %), birds (22.9 %), anthropogenic items (16 %), ungulates (4.7 %) and small carnivores (4.2 %). Remains of undetermined mammals were found in 62 stomachs (43.1 %) (Table 1).

**Table 1:** Frequency in non-empty stomachs (FO%) and mean ± standard error of abundance (both in ml and %) of different items in the diet of red foxes from the Province of Bolzano, northeastern Italy, after gastric content analysis. Items are part of the category mentioned above if written either not in capital letters or right adjusted. aRodent prey which were genetically identified to species are listed in Table 2.

CATEGORY	ITEM	N STOMACHS	FO (%)	MEAN VOLUME (ml)	MEAN VOLUME (%)
PLANT ITEMS		128	88.9	23.4 ± 2.8	33.6 ± 2.6
	FRUIT	14	9.7	12.7 ± 4.5	14.7 ± 5.6
	*Vaccinium myrtillus*	2	0.7	48.8 ± 44.9	34.8 ± 32.8
	*Fragaria* sp.	2	1.4	29.1 ± 9.8	35.8 ± 29.1
	*Prunus* sp.	5	3.4	10.3 ± 7.1	7.8 ± 3.9
	*Prunus avium*	2	1.4	22.4 ± 16.1	8.2 ± 6.3
	*Malus domestica*	6	4.1	3.1 ± 1.2	5.7 ± 4.8
	*Pyrus communis*	2	1.4	0.9 ± 0.6	0.5 ± 0
	*Vitis vinifera*	1	0.7	4.8	3
	*Fraxinus* sp.	2	1.4	0.5 ± 0	0.5 ± 0
	*Leucanthemum vulgare*	1	0.7	1.5	10
	GRASS	123	85.4	20.3 ± 2.6	30.4 ± 2.6
SMALL MAMMALS		47	32.6	65.3 ± 8.9	62.6 ± 5
	RODENTS^a^	46	31.9	66.6 ± 9	63.3 ± 5
	*Marmota marmota*	1	0.7	5	10
	INSECTIVORES	1	0.7	8.4	27.6
	*Erinaceus europaeus*	1	0.7	8.4	27.6
UNGULATES		7	4.7	133.6 ± 99.4	42.4 ± 13.6
	*Capreolus capreolus*	2	1.4	20.7 ± 18.2	10 ± 5
	*Cervus elaphus*	1	0.7	31.5	45
SMALL CARNIVORES		6	4.2	23.9 ± 7.6	30.6 ± 10.9
	*Meles meles*	1	0.7	34.5	69
	*Martes foina*	1	0.7	48	21
	*Martes martes*	1	0.7	38.5	14
	*Felis silvestris silvestris*	1	0.7	6	60
UNDETERMINED MAMMALS		62	43.1	37.6 ± 7.7	52.3 ± 4.2
INVERTEBRATES		40	27.8	27.5 ± 7.3	23.7 ± 4.1
	ARTHROPODA	39	27.1	8.8 ± 3.6	9.6 ± 2.7
	Coleoptera	13	9	7.8 ± 4.3	10 ± 4.2
	Hymenoptera	7	4.9	20.3 ± 10.7	17.1 ± 5.8
	EARTHWORMS	20	13.9	36.3 ± 11.1	26.2 ± 5.3
BIRDS		33	22.9	39.5 ± 8.6	32.1 ± 5.3
	*Gallus gallus*	8	5.6	49 ± 13.7	37.4 ± 5.9
	Corvids	1	0.7	2	1
ANTHROPOGENIC ITEMS		23	16	65.6 ± 16.8	37.7 ± 6.1
	Plastic-artificial objects	12	8.3	14.5 ± 5.7	10.8 ± 2.2
	Human food	18	12.5	73.6 ± 22.9	39.5 ± 8.6
UNDEFINED		16	11.1	32.7 ± 18	20.4 ± 5.5

a Rodent prey species which were molecularly identified by PCR are listed in Table 2.

Most prey rodents were partially digested, but nine stomachs contained 22 rodent carcasses with intact internal cavities. Stomachs contained from one to 17 individual rodents with a mean of 3.3 ± 0.6 SE carcasses per stomach (median = 2).

Of note, ascarids were opportunistically reported from the gastric content of nine stomachs, with a mean ± SE of specimens of 3.3 ± 1.41.

### 3.1 Prey species assessment

Molecular identification identified a minimum of 142 out of 143 rodent prey to species (‘MNI’), from a total of 42 stomachs (29.2 %). Abundance and frequency of rodents are listed in Table 2; the two species considered the most important IH for *Em* life cycle in Europe (Beerli et al., 2017) are written in bold. Co-predation on more than one rodent species occurred in six foxes.

**Table 2:** Species, minimum number of individuals (MNI), occurrence (number of stomachs and % of Frequency of Occurrence, FO) and volume (mean ± standard error) of rodent prey that were genetically identified. The two key intermediate host species for *Em* in Europe are in bold. Intermediate host (IH) competence: 2 = Competent species; 1 = Susceptible species; 0 = Resistant species; *ni* = not investigated. FO% is calculated on the total non-empty stomachs (144).

RODENT SPECIES	MNI	N STOMACHS^a^	FO (%)	MEAN VOLUME (ml)	MEAN VOLUME (%)	IH COMPETENCE
***Microtus arvalis***	116	30	20.8	66.1 ± 10.6	65.4 ± 5.4	2
*Microtus lavernedii*	5	1	0.7	73.8	56.8	2
*Microtus liechtensteini*	1	1	0.7	14.9	11.3	*Ni*
*Microtus subterraneus*	2	2	1.4	18.1 ± 1.9	55.8 ± 44.2	1
*Clethrionomys glareolus*	6	4	2.8	34.3 ± 6.6	19.8 ± 6.5	2
***Arvicola amphibius***	9	8	5.6	45.2 ± 20.9	56.3 ± 14.2	2
*Apodemus sylvaticus*	2	1	0.7	45	45	2
*Apodemus flavicollis*	1	1	0.7	16.5	6.1	1

a Number of stomachs in which each single species was found in 42 of a total of 144 non-empty stomachs.

As for the proportion of IHs in the diet, we detected predation of seven rodent species which are known to be susceptible to *Em* infection, namely the common vole *M. arvalis*, the water vole *Arvicola amphibius*, the Mediterranean field vole *M*. *lavernedii*, the European pine vole *M*. *subterraneus*, the bank vole *Clethrionomys glareolus*, the yellow-necked mouse *Apodemus flavicollis*, and the wood mouse *Ap*. *sylvaticus* (Fig. 1). Their MNI (and FO%) were as the following: 116 *M. arvalis* (20.8), nine *A. amphibius* (5.6), five *M. lavernedii* (0.7), two *M. subterraneus* (1.4), six *C. glareolus* (2.8), one *Ap. flavicollis* (0.7) and two *Ap. sylvaticus* (0.7) (Table 2). Moreover, we detected predation upon one Liechtenstein's pine vole *M*. *liechtensteini* (FO 0.7 %), whose susceptibility status for *Em* has never been investigated.

Overall, competent intermediate hosts were found in 40 out of 144 stomachs (27.8 %), and 97.2 % of individuals classified to species were competent IH species (138/142). *M. arvalis* represented the species most commonly found in the stomachs, and also the most preyed on species in terms of number of individuals (116/142 or 81.7 % of all individuals identified to species).

### 3.2 *Echinococcus multilocularis* infection

No *Em* metacestodes or protoscoleces were detected under a light microscope from the residuals of 136 stomachs. None of the collected internal organs from prey rodents showed macroscopically visible lesions of *Em* under a stereomicroscope.

We performed both RT-PCR and cPCR on 43 livers, 13 thoracic contents (lungs and/or heart), 10 kidneys and 45 ‘NA’ (not assigned or unrecognisable abdominal content) from a total of 97 rodents. The RT-PCR detected *Em* DNA in one out of 38 (2.6 %) examined livers of the common vole *M. arvalis*, with a mean Ct value of 38.8 (38.225 and 39.376). Instead, cPCR detected *Em* DNA in this same sample and in one additional analysed liver of *M. arvalis* (2/38, 5.3 %) (Fig. 1), but also in the heart and lungs of a third common vole. Interestingly, these additional two positive individuals were sampled from a single stomach of another *Em*-infected red fox. Therefore, *Em* apparent prevalence in *M. arvalis* was estimated at 0.026 (95 % CI: 0.003–0.116) and at 0.053 (95 % CI: 0.005–0.182) by RT-PCR and cPCR, respectively, if considering only livers. Apparent prevalence was 0.035 when considering all common voles regardless the examined tissues (3/85; 95 % CI: 0.008–0.103). None of the internal organs from the other rodent species were RT-PCR or cPCR *Em*-positive.

As for the apparent prevalence of the other species with *Em*-negative results, the upper limits of the 95 % confidence intervals were 0.292 (0/7 *A. amphibius*) and 0.667 (0/2 *M. subterraneus*); such upper limit could not be estimated for species with only one individual with available tissue for running *Em* DNA investigation (*M. lavernedii*, *M. liechtensteini*, *C. glareolus*).

## 4 Discussion

We investigated the frequency of IHs in the stomachs of red foxes to estimate *Em* transmission risk to DHs inside a locally persistent autochthonous focus in a marginal endemic region of the Italian Alps. Rodents were commonly found, but were not the most prevalent dietary item in this area. Almost all of the rodent prey individuals were of species known to be competent or susceptible IHs for *Em* (Romig and Wassermann, 2024). Interestingly, the common vole was the most frequently prey IH, representing 81.7 % of the total rodent individuals found in the red fox stomachs and the only *Em*-positive IH found in our study, suggesting a key role of this species in transmission of *Em* in this area. Notably, the three *Em*-positive common voles were found in the stomachs of two *Em*-positive red foxes.

This study did not target the dietary spectrum in general but focused on small mammals as a target of *Em* infection; therefore, less attention was given to species characterisation of other item categories, and we could not directly compare these results with the dietary habits of red foxes from other European regions (Soe et al., 2017). For example, *A. amphibius* and *Ap. sylvaticus*, which have previously been found to be *Em*-positive from liver-DNA amplification studies in the same Province (Sgubin et al., 2022), were not common in the diet of red foxes in our study. Presumably, *M. arvalis* is central to *Em* transmission in this area, likely because of its high frequency in the diet of the main DH in this southern marginal area of the *Em* European distribution.

The three *Em*-positive common voles did not present any visible organ lesion that could be attributed to the infections (e.g. vesicles). This might be due an early abortive infection, or simply because metacestodes and protoscoleces development requires up to four months (Woolsey et al., 2015, 2016). This result is not unusual, as several studies also described a higher prevalence of *Em* detected by DNA amplification than by morphological and histopathological approaches (Stieger et al., 2002; Reperant et al., 2009; Massolo et al., 2021). However, to the best of our knowledge, only two studies investigated *Em* infection in rodent livers with no macroscopic lesions (Beiromvand et al., 2013; Massolo et al., 2021), whereas the vast majority of studies applied a molecular approach only to confirm clear or suspicious lesions (Oksanen et al., 2016; Cartuyvels et al., 2022; Martini et al., 2022; Gürler et al., 2023). The infection rate from this study could also be considered conservative, since the number of undigested or partially digested rodents with enough preserved internal tissues was low. Many rodents were shrivelled, as the digestive process had already started. Out of 85 common voles with sampled tissues for *Em* screening, livers collection was possible for only 38 individuals, of which only two had clearly distinguishable lobes. So the lack of detectable lesions could also be due to the poor tissue conditions.

Prevalence of *Em* in rodents is known to be generally low (Oksanen et al., 2016; Romig et al., 2017), but *Em* transmission in low prevalence conditions might be facilitated by higher predation risk of infected IHs (Raoul et al., 2010, 2015; Martini et al., 2024), persistence of eggs under suitable environmental factors (Veit et al., 1995), and short-term larval maturation (Woolsey et al., 2015, 2016). Locally, infection in IHs can be as high as 50 % (Romig et al., 2017), although such levels are unusual.

Intermediate hosts normally show significantly lower infection rates than coexisting DHs, and this is true for *Em*: competent small mammals usually have much lower infection rates than wild canids, as the likelihood of encounter with infectious parasitic propagules (eggs) is much lower than those of DHs (infective prey), and likely also because of the high virulence of *Em* in IHs that increases their mortality, and possibly of the parasitic manipulation that facilitates their predation and thus removal from the population (Hofer et al., 2000; Miller et al., 2016; Beerli et al., 2017; Martini et al., 2024). Therefore, our results are consistent with the most recent prevalence estimates in red foxes in the same area (Obber et al., 2022).

However, by sampling from the stomach of definitive hosts we achieved two objectives: on one hand, by assessing the richness and relative abundance of potential IHs in the diet of the DH, we estimated the frequency of encounter and exposure of the DH to potentially infected IHs. On the other hand, we also note that, by comparing the data on infected species and prevalence in IH prey measured here to those obtained by direct sampling of IHs in the same area, this would allow an estimate of the effects of the theorized parasitic manipulation of IH behaviour, allegedly affecting predation risk and thus facilitating transmission (Martini et al., 2024).

## 5 Conclusions

Our results showed that sampling IH prey from the stomach of DHs is a valid alternative to direct sampling of IHs to investigate eco-epidemiological questions related to *Em* in marginal endemic areas, where prevalence is relatively low. Protocols for sampling carcasses and stomach contents of DHs should be improved to include more seasons and increase sample size for statistical analyses. Future studies should focus on the relevance of various small mammal species as IHs of *Em* in other marginal regions where prior information is missing or is being collected. This could help to build transmission and risk models to be validated for such areas and, in general, for other complex-life cycle pathogens transmitted through a predator-prey cycle.

## CRediT authorship contribution statement

**Cafiero Salvatore Andrea:** Writing – original draft, Methodology, Investigation. **Cenni Lucia:** Methodology. **Rossi Chiara:** Supervision, Methodology. **Pascoe Emily Louise:** Investigation, Methodology, Writing – review & editing. **Obber Federica:** Methodology, Investigation. **Da Rold Graziana:** Methodology, Investigation. **Citterio Carlo Vittorio:** Resources, Supervision. **Casulli Adriano:** Writing – review & editing. **Hauffe Heidi Christine:** Supervision, Methodology, Conceptualization. **Massolo Alessandro:** Writing – review & editing, Supervision, Conceptualization.

## Funding sources

This research was supported by the Ministry of University and Research (MUR), Decreto Ministeriale 1061/2022, through the National Operational Programme on Governance and Institutional Capacity (PON), one of the Cohesion Policy instruments (2014-2020 cycle: Research and Innovation resources - Green/Innovation Action), financed by the European Union through the European Structural and Investment (ESI) Funds. This research was partially supported by the European Commission's 10.13039/501100007561Directorate-General for Health and Food Safety (DG SANTE) under the grant agreement no. 101144113: “Work programme 2023–2024 of EU European Reference Laboratory for the Parasites (EURL-P)”. This work was partly funded also by the Italian Ministry of Health (project codes: IZSVe RC 05/2019).

## Declaration of interest

All authors declare that there are no competing interests.
